# The Human Brain Project—Synergy between neuroscience, computing, informatics, and brain-inspired technologies

**DOI:** 10.1371/journal.pbio.3000344

**Published:** 2019-07-01

**Authors:** Katrin Amunts, Alois C. Knoll, Thomas Lippert, Cyriel M. A. Pennartz, Philippe Ryvlin, Alain Destexhe, Viktor K. Jirsa, Egidio D’Angelo, Jan G. Bjaalie

**Affiliations:** 1 Institute for Neuroscience and Medicine (INM-1), Forschungszentrum Jülich, Germany; 2 C. and O. Vogt Institute for Brain Research, University Hospital Düsseldorf, Heinrich Heine University Düsseldorf, Düsseldorf, Germany; 3 Institut für Informatik VI, Technische Universität München, Garching bei München, Germany; 4 Jülich Supercomputing Centre, Institute for Advanced Simulation, Forschungszentrum Jülich, Germany; 5 Swammerdam Institute for Life Sciences, Faculty of Science, University of Amsterdam, the Netherlands; 6 Department of Clinical Neurosciences, Centre Hospitalo-Universitaire Vaudois (CHUV) and University of Lausanne, Lausanne, Switzerland; 7 Unité de Neurosciences, Information & Complexité (UNIC), Centre National de la Recherche Scientifique (CNRS), Gif-sur-Yvette, France; 8 Institut de Neurosciences des Systèmes, Inserm UMR1106, Aix-Marseille Université, Faculté de Médecine, Marseille, France; 9 Department of Brain and Behavioral Science, Unit of Neurophysiology, University of Pavia, Pavia, Italy; 10 Institute of Basic Medical Sciences, University of Oslo, Oslo, Norway

## Abstract

The Human Brain Project (HBP) is a European flagship project with a 10-year horizon aiming to understand the human brain and to translate neuroscience knowledge into medicine and technology. To achieve such aims, the HBP explores the multilevel complexity of the brain in space and time; transfers the acquired knowledge to brain-derived applications in health, computing, and technology; and provides shared and open computing tools and data through the HBP European brain research infrastructure. We discuss how the HBP creates a transdisciplinary community of researchers united by the quest to understand the brain, with fascinating perspectives on societal benefits.

## Abstract 2

This Community Page article presents the Human Brain Project; a European Flagship project with a ten-year horizon aiming to understand the human brain and translate neuroscience knowledge into medicine and technology.

## Introduction

To decode the multilevel brain's complexity, the Human Brain Project (HBP) combines empirical neuroscience in the human brain and in animals with theory and modeling, relying on and developing advanced information and communication technology (ICT) including computing, big data analytics, artificial intelligence (AI), and simulation [1]. The project represents a large-scale, interdisciplinary approach and consists of 12 subprojects—i.e., mouse brain organization, human brain organization, systems and cognitive neuroscience, theory, neuroinformatics, brain simulation, medical informatics, high-performance analytics and computing, neuromorphic computing, neurorobotics, administrative support, and ethics and society. Knowledge and constraints in neuroscience are drivers for developing research platforms through a codesign process. This has proven to be a very successful approach for transdisciplinary work in the HBP. Through codesign, the HBP is developing and releasing a unique European brain research infrastructure. To enable neuroethical analysis and develop, broaden, and enhance responsible research and innovation, the HBP was one of the first initiatives worldwide to establish a dedicated subproject for ethics.

## Neuroscience: Understanding the brain at multiple scales

The multiscale approach of the HBP links experiments conducted at the molecular, subcellular, and cellular levels and at the level of neuronal populations and macroscopic regions, up to large-scale networks and behavior. Research on mouse brain organization primarily gathers structural and physiological data at the subcellular and cellular levels, which is critical for biophysically detailed modeling and simulations. For instance, novel data on microcircuits in the cerebellum shed new light on its function, including forward and feedback control [2].

**Fig 1:**
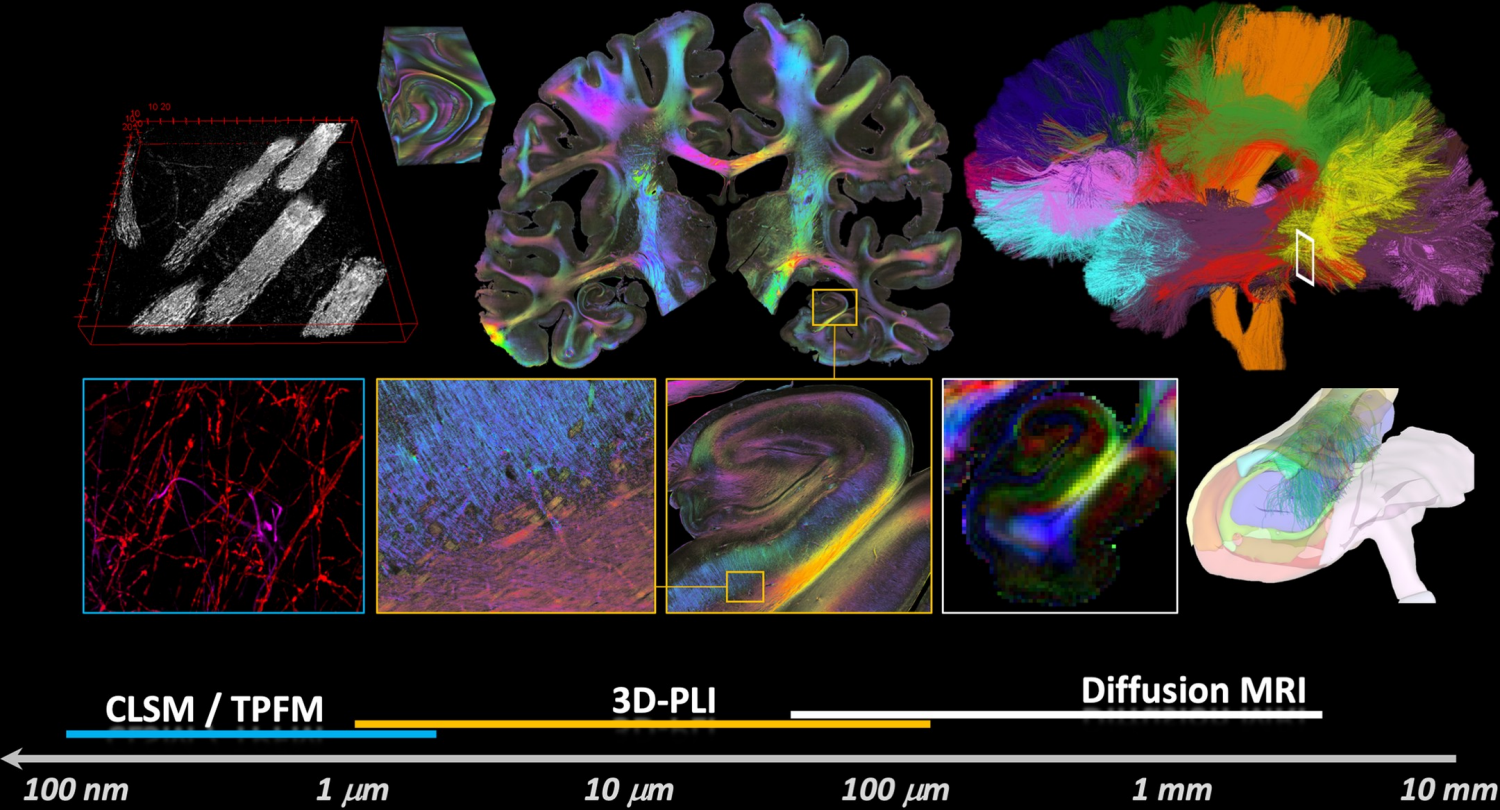
Multiscale organization of brain connectivity. Different methods are being used in the HBP to analyze neuronal connections from the nanometer scale to the centimeter scale. Although TEM/SEM (e.g., Rodriguez-Moreno and colleagues [5]) and CLSM/TPFM (e.g., Silvestri and colleagues [6]) can image subcellular structures, including synapse, with great detail, they cannot cover the whole human brain. 3D-PLI has a spatial resolution down to 1.3 μm, which resolves most of the myelinated fibers, and has the potential to image the whole human brain (e.g., Axer and colleagues [7]). Diffusion MRI is an in vivo method covering the whole brain, but with limited spatial resolution, which does not resolve single nerve fibers (e.g., Beaujoin and colleagues [8]). The possibility to link these different data shows the great advantage of HBP—it facilitates the combination of approaches at scale, backed by high-performance computing and the data exchange infrastructure FENIX. CLSM, confocal laser scanning microscopy; FENIX, Federated Exascale Network for data Integration and eXchange; HBP, Human Brain Project; PLI, polarized light imaging; TEM, transmission electron microscopy; TPFM, two-photon fluorescence microscopy. *Figure elements provided by Markus Axer*.

Electron microscopy, two-photon and light-sheet imaging, polarized light imaging (PLI), and diffusion MRI, along with advanced electrophysiological techniques including patch clamping and multielectrode array recordings in vitro and in vivo as well as electroencephalography (EEG), provide data on the connectome on the different spatial and temporal scales (Fig 1). These data, together with those on the cellular and molecular organization, functional and connectivity data, and many more, are integrated into the HBP Brain Atlases, which provides a basis for human and rodent brain research [3]. The atlases also establish the structural frame for modeling and simulation studies, which is a unique feature of the HBP approach. For example, simulations use patient-specific brain connectivity and imaging of malformations to personalize network models for seizure prediction and surgery [4].

Understanding the brain at multiple scales requires the integration of anatomy and physiology with cognitive and systems neuroscience to flesh out the neural mechanisms underlying cognition—e.g., in object recognition, visuomotor control, episodic memory, sleep, wakefulness, and consciousness. To this end, researchers combine experimental studies in rodents and humans, integrate data in computational models and simulations of large-scale neuronal networks, and simplify and apply these models to control physical robots or simulated agents in order to capture essential features of animal and human behavior. Neuroscientists working on the cellular or molecular level exchange results with researchers working in cognitive and systems as well as theoretical neuroscience to cast neurobiological principles into new theories and formal models, which then prompt simulation studies and the development of neuromorphic computing systems. There is still no comprehensive theory describing information processing in the brain, but important building blocks can be won through these approaches and may constitute the basis for such a theory in the future.

## The European brain research infrastructure

**Fig 2:**
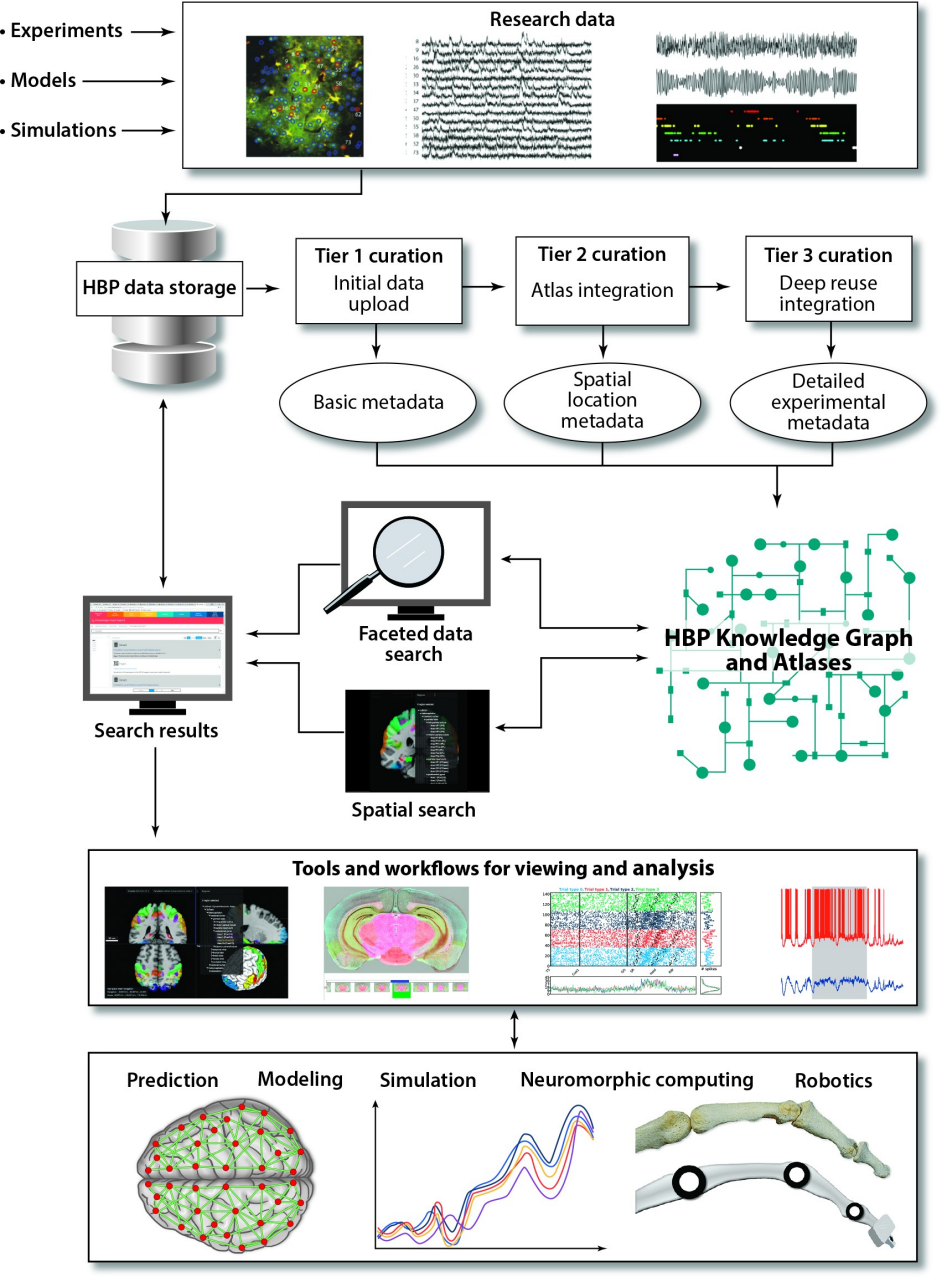
The HBP integrated platform. Research data from experiments, models, or simulations are uploaded to data storage at the HBP high-performance computing centers. The data are tagged with metadata through a 3-tier curation process covering basic metadata, metadata connecting the data to the HBP Atlases, and additional detailed metadata depending on the data modality. The data are made accessible for users through searches for metadata (“Faceted data search” or “Spatial search”) in the online HBP Knowledge Graph and HBP Atlases. The “Search results” give access to collections of data accompanied by information about the project they belong to, the experimental methods used to produce the data, the terms of use, and the citation requirements for use of the data. In the present implementation, users can (1) download the data, (2) inspect the data using an online atlas viewer or a virtual microscopy tool showing image data with atlas overlays and spatial coordinates, or (3) analyze the data using the “Tools and workflows for viewing and analysis” accessible through the HBP Collaboratory, the portal to the use of HBP tools and services, or directly through the atlas for some cases. The HBP Knowledge Graph and HBP Atlases are openly available at https://www.humanbrainproject.eu/en/explore-the-brain/. Accounts for use of the HBP Collaboratory can be requested at https://services.humanbrainproject.eu/oidc/login. HBP, Human Brain Project. *Image credits*: *doi*:*10*.*1371/journal*.*pone*.*0118277*, *doi*:*10*.*1371/journal*.*pbio*.*1002383*, *doi*:*10*.*1371/journal*.*pbio*.*1000173 (based on a dataset available at doi:10.12751/g-node.f83565)*.

This infrastructure consists of different components—neuroinformatics, simulation, high-performance analytics and computing, neuromorphic computing, and neurorobotics. To enable interdisciplinary research, a seamless transition and integration of the different components and data is necessary. Collaborating with the neuroscience branches, the HBP is developing and providing workflows, from organizing and storing data and making them accessible according to the Findable Accessible Interoperable Reusable (FAIR) data principles [9] to data analysis, modeling, simulation, and brain-inspired technologies (Fig 2).

In addition, the voucher system of the HBP allows researchers outside the project to request innovation support or technical support for the modification of tools and workflows to meet their needs, thus facilitating broader uptake of the integrated platform services.

The High-Level Support Team (HLST) of the HBP is available for answering questions or resolving issues with the use of the tools and services and for adapting tools and workflows for specific use cases. Specifically, researchers outside HBP can make their data discoverable and accessible in the Knowledge Graph and Atlases by applying for access to curation services and data storage through the HLST. The HLST help desk and ticketing system is available at the following e-mail: support@humanbrainproject.eu.

Various types of neuroscience data are being generated, with differences in format, metadata, or level of precision. Through a curation process, metadata are assigned and stored in the HBP Knowledge Graph, an online graph database allowing advanced searches (Fig 2). Thereby, data and models become findable and interpretable. Data can be downloaded or used directly in workflows available through the computing infrastructure, with terms of use and licenses defined for all available data sets. Data are also being made available through the Knowledge Graph with integrated multilevel HBP Atlases, holding information about the brain in standard reference spaces. The Human Brain Atlas supports different reference spaces including the T1-weighted, single-subject template of the Montreal Neurological Institute (MNI) [10] and the BigBrain [11]. The atlases are more and more populated with data from the different fields to form fully interoperable, multimodal representations of the human and rodent brain. They are compatible with other resources [12] and capable of integrating data not only from healthy subjects but also from patients.

The Medical Informatics Platform (MIP) exploits new ICT in the context of clinical neurosciences [13]. It combines local components installed in every participating hospital and a web-based central node connecting the local components. Its architecture enables the extraction, processing, and analysis of medical data collected during either clinical practice or research. The MIP allows the automatic generation of brain regional volumes from T1 MRI data. The key is to enable General Data Protection Regulation (GDPR)-compliant federated analysis of distributed clinical datasets without moving the data out of the hospital, a crucial issue in the context of data privacy concerns and regulations. This unique feature opens the way for studies of unmatched scale, keeping in mind that 165 million European citizens suffer from brain diseases. A first proof-of-concept study in the field of dementia collected >6,000 datasets from three hospitals and analyzed them together with data from Alzheimer’s Disease Neuroimaging Initiative (ADNI). The future MIP will include many more hospitals and consider data from patients with epilepsy, traumatic brain injury, and behavioral disorders. It offers an ideal infrastructure to implement AI-based diagnostic and patient management tools for clinical practice in conjunction with modeling and simulation efforts in personalized medicine.

Some of the research data in brain science already reach the petabyte range. The HBP storage at high-performance computing centers in Europe, the FENIX infrastructure, enables researchers to upload their data and use high-end simulation and processing of big data in an easy and straightforward way from wherever they are. To bring neuroscientists to supercomputing is one of the aims of the HBP; it opens new ways to solve neuroscientific questions requiring significant compute resources. Computer specialists benefit from neuroscience use cases, which are instrumental to identify the characteristics of future, extreme-scale computers including modular supercomputers. Already, the integrated platform of the HBP is the first research infrastructure that is embedding two worldwide, unique neuromorphic computing machines with novel non–von Neumann computing architecture (SpiNNaker and BrainScaleS) into user workflows [14]. This powerful computing infrastructure will smoothly handle complex workflows, combining, for example, compute-intensive simulation with the analysis of data using deep learning.

Neurorobotics is a brain-inspired technology; a novel interdisciplinary field of science at the confluence of neuroscience, robotics, and artificial intelligence; and part of the research infrastructure. It recognizes the fact that the brain is embedded into a body and that this body is itself embedded into a dynamic environment. This approach considers that environmental interactions cannot be ignored to faithfully simulate the brain and understand behavior. This embodiment (through either a simulated agent or a physical robot) provides neuroscientists with a new experimental paradigm to test their neural models [15]. Simulations implementing closed-loop experiments in neurorobotics encompass the full action–perception–cognition loop. Observation of brain activity and physical performance in the context of relevant behavioral tasks then enables researchers to either refute or support the theory behind their neural models, thus uniquely informing their scientific investigations. This line of research will eventually lead to a new paradigm, “closed-loop neuroscience,” for which the HBP intends to provide the necessary infrastructure. Such an example illustrates the synergistic relationship between neuroscience, ICT, and brain-like computing within HBP.

The brain research infrastructure will unify the individual components into one cloud-based superstructure, facilitating the exchange of knowledge, data, models, and algorithms within HBP as well as between the HBP and the “outside world.” Research infrastructures in other fields, such as particle physics, served as role models to create active, tangible interfaces between researchers and the infrastructure.

## Concluding remarks

The HBP is pursuing an open-science approach. Simulation engines, models, analysis tools, data, and the HBP Atlases are shared with the community through a web-based system as a common entry point. Multilevel brain complexity is a challenge that needs a sustainable and strong—but also flexible—research infrastructure at the interface of neuroscience and computing, which is developed by the project. It offers and develops services to the research community to solve scientific problems from various fields including FAIR data; atlasing; medical brain activity data; web-based, interactive supercomputing; closed-loop neuroscience; robotics and AI; and modeling and simulation workflows. The HBP approach has already led to a significant number of breakthroughs, among them the development of novel spike-based learning algorithms, which were implemented on neuromorphic computers with the aim of obtaining general-purpose tools for a new generation of AI applications; innovative theoretical models that made use of results from experimental neuroscience addressing the multiscale organization of the brain; and significant advances in understanding the neural basis of learning and perception [16], spatial memory [17], multisensory integration [18], and sleep and consciousness [19]. These are a few examples illustrating that the integration of neuroscience and ICT within a common framework opens new ways toward a better understanding of brain complexity. (For a regularly updated overview, see https://www.humanbrainproject.eu/en/science/highlights-and-achievements/.)

A research culture of collaboration and data sharing, which is accompanied by activities addressing ethical and philosophical issues and their societal implications, has been key for the HBP from the very beginning. The HBP infrastructure provides a concrete basis to collaborate with the wider, international research community. It is linking the European project into an international context—e.g., through the International Brain Initiative (IBI). The IBI was created in 2017 with the Australian Brain Alliance, Japan Brain Mapping by Integrated Neurotechnologies for Disease Studies (Brain/MINDS) Project, Korea Brain Initiative, the European HBP, and the United States Brain Initiative as the initial members. It has the aim of coordinating efforts between the global initiatives to speed up progress on decoding the brain’s code.

## Abbreviations

ADNI: Alzheimer’s Disease Neuroimaging Initiative
AI: artificial intelligence
Brain/MINDS: Brain Mapping by Integrated Neurotechnologies for Disease Studies
CLSM: confocal laser scanning microscopy
EEG: electroencephalography
FAIR: Findable Accessible Interoperable Re-usable
FENIX: Federated Exascale Network for data Integration and eXchange
GDPR: General Data Protection Regulation
HBP: Human Brain Project
HLST: High-Level Support Team
IBI: International Brain Initiative
ICT: information and communication technology
MIP: Medical Informatics Platform
MNI: Montreal Neurological Institute
PLI: polarized light imaging
TEM: transmission electron microscopy
TPFM: two-photon fluorescence microscopy

## References

[1] Amunts K, Ebell C, Muller J, Telefont M, Knoll A, Lippert T. The Human Brain Project: Creating a European Research Infrastructure to Decode the Human Brain. Neuron. 2016;92(3):574–81. 10.1016/j.neuron.2016.10.046 27809997

[2] D'Angelo E, Wheeler-Kingshott CG. Modelling the brain: Elementary components to explain ensemble functions. Riv Nuovo Cimento. 2017;40(7):297–333. 10.1393/ncr/i2017-10137-5 WOS:000406931800001.

[3] Bjerke IE, Øvsthus M, Papp EA, Yates SC, Silvestri L, Fiorilli J, et al Data integration through brain atlasing: Human Brain Project tools and strategies. Eur Psychiatry. 2018 [cited 2019 Jun 25];50:70–6. Available from: 10.1016/j.eurpsy.2018.02.004 29519589

[4] Proix T, Bartolomei F, Guye M, Jirsa VK. Individual brain structure and modelling predict seizure propagation. Brain. 2017;140:641–54. 10.1093/brain/awx004 WOS:000397317100021. 28364550 PMC5837328

[5] Rodriguez-Moreno J, Rollenhagen A, Arlandis J, Santuy A, et al Quantitative 3D ultrastructure of thalamocortical synapses from the “lemniscal” ventral posteromedial nucleus in mouse barrel cortex. Cerebral Cortex. 2018; 28(9): 3159–3175. 10.1093/cercor/bhx187 28968773 PMC6946031

[6] Silvestri L., Allegra Mascaro AL, Costantini I, Sacconi L, Pavone FS. Correlative two-photon and light sheet microscopy. Methods. 2014; 66(2): 268–272. 10.1016/j.ymeth.2013.06.013 23806642

[7] Axer M, Strohmer S, Gräßel D, Bücker O, Dohmen M, Reckfort J, Zilles K, Amunts K. Estimating fiber orientation distribution functions in 3D-Polarized Light Imaging. Front. Neuroanat. 2016; 10:40 10.3389/fnana.2016.00040 27147981 PMC4835454

[8] Beaujoin J, Palomero-Gallagher N, Boumezbeur F, Axer M, Bernard J, Poupon F, Schmitz D, Mangin J-F, Poupon C. Post-mortem inference of the human hippocampal connectivity and microstructure using ultra-high field diffusion MRI at 11.7 T. Brain Structure and Function. 2018; 223(5):2157–2179. 10.1007/s00429-018-1617-1 29387938 PMC5968081

[9] Wilkinson MD, Dumontier M, Aalbersberg IJJ, Appleton G, Axton M, Baak A, et al The FAIR Guiding Principles for scientific data management and stewardship. Scientific data. 2016;3:160018 10.1038/sdata.2016.18 . 26978244 PMC4792175

[10] Evans AC, Janke AL, Collins DL, Baillet S. Brain templates and atlases. Neuroimage. 2012;62(2):911–22. 10.1016/j.neuroimage.2012.01.024 22248580

[11] Amunts K, Lepage C, Borgeat L, Mohlberg H, Dickscheid T, Rousseau ME, et al BigBrain: An ultrahigh-resolution 3D human brain model. Science. 2013;340(6139):1472–5. 10.1126/science.1235381 23788795

[12] Amunts K, Hawrylycz M, Van Essen D, Van Horn JD, Harel N, Poline JB, et al Interoperable atlases of the human brain. Neuroimage. 2014;99:525–32. 10.1016/j.neuroimage.2014.06.010 24936682

[13] Melie-Garcia L, Draganski B, Ashburner J, Kherif F. Multiple linear regression: Bayesian inference for distributed and Big Data in the Medical Informatics Platform of the Human Brain Project. BioRxiv [Preprint]. 2018 [cited 2019 Jun 25]. Available from: https://www.biorxiv.org/content/10.1101/242883v1.

[14] van Albada SJ, Rowley AG, Senk J, Hopkins M, Schmidt M, Stokes AB, et al Performance comparison of the digital neuromorphic hardware SpiNNaker and the neural network simulation software NEST for a full-scale cortical microcircuit model. Front Neurosci. 2018;12 10.3389/fnins.2018.00291 WOS:000432839000001. 29875620 PMC5974216

[15] Knoll A, Gewaltig M-O. Neurorobotics: A strategic pillar of the Human Brain Project. The intersection of robotics and neuroscience. Science/AAAS. 2016: 25–34.

[16] Takahashi N, Oertner TG, Hegemann P, Larkum ME. Active cortical dendrites modulate perception. Science. 2016;354(6319):1587–90. 10.1126/science.aah6066 28008068

[17] Bos JJ, Vinck M, van Mourik-Donga LA, Jackson JC, Witter MP, Pennartz CMA. Perirhinal firing patterns are sustained across large spatial segments of the task environment. Nature communications. 2017;8 10.1038/ncomms15602 WOS:000402047500001. 28548084 PMC5458559

[18] Gentile F, van Atteveldt N, De Martino F, Goebel R. Approaching the ground truth—Revealing the functional organization of human multisensory STC using ultra high field fMRI. J Neurosci. 2017 10.1523/jneurosci.0146-17.2017 . 28912157 PMC6596547

[19] Storm JF, Boly M, Casali AG, Massimini M, Olcese U, Pennartz CMA, et al Consciousness Regained: Disentangling Mechanisms, Brain Systems, and Behavioral Responses. J Neurosci. 2017;37(45):10882–93. 10.1523/JNEUROSCI.1838-17.2017 WOS:000414662100014. 29118218 PMC5678021

